# Supplementary material for: Learning curve and surgical outcome of robotic assisted colorectal surgery with ERAS program
Source: Sci Rep. 2022 Nov 29;12:20566. doi: 10.1038/s41598-022-24665-w (PMC9709162; doi:10.1038/s41598-022-24665-w)
Supplement: Supplementary file 1 — Supplementary Information 1. [file 41598_2022_24665_MOESM1_ESM.zip › LSC Raw data-Ñ[▒K/052/HS_ñw╝╨░O▒Kñσ.pdf]

正式摘要(電子簽章：00000178865398)

W95 / W95 ☐ 英文標題(注意:無英文姓名-請補病人基本檔!) ☐【讀取】憑證

臺中榮民總醫院

出院病歷摘要

轉入號：　轉出號：

[redacted]

**(8)**流水編號	
**(9)**入院日期	108/10/17 (2019) 直腸外科 W95 - 09
**(10)**轉科日期	
**(11)**出院日期	108/10/25 (2019) 住院天數計 8 日

診斷

- 入院 :Rectal cancer, s/p CCRT under Gy / 5 fr by Tomotherapy and Ufur on 2019/08
- 出院 :Rectal cancer, s/p CCRT under Gy / 5 fr by Tomotherapy and Ufur on 2019/08 - s/p Laparoscopic assisted low anterior resection with end-to-end anastomosis on 2019/10/18

主訴

- rectal cancer found at health checkup

病史

- This 54 y/o man was healthy before.
  He was told to have stool occult blood positive at health check up at 署立南投醫院。Colonoscopy showed rectal cancer.
  He denied any other discomfort, denied body weight loss. Therefore he went to our Hospital for help. CCRT was suggested. The patient was performed CCRT under Gy / 5 fr by Tomotherapy and Ufur. This time he was amditted for surgical treatment

體檢發現

- Anal inspection:
  Recto digital examination:PALAPBEL TUMOR, ANTERIOR ....
  - General appearance :
    - easy looking , conscious clear , without cardiopulmonary distress
    - Vital signs自動帶入date 體溫(BT) 脈搏(P) 呼吸(R) 血壓(BP) 身高(BH) 體重(BW) BMI
      20191017 36℃ 86次/分 19次/分 123/78mmHg 165.3cm 76.7kg 28.1
    - Vital signs: B.P. 123 mmHg , P.R. 86 /min , R.R. 19 /min , B.T. 36 ℃
  - HEENT :
    - Eyes: no anemic conjunctiva, no icteric sclera, normal EOM
    - ENT: no otorrhea, no rhinorrhea, no erythematous mucosa, no injected tonsil, no deformity
  - Neck: supple, freely movable, trachea midline,no deformity, no engorged jugular vein , no palpable thyroid or lymph node enlargement
  - Chest:
    - Inspection: Grossly normal, symmetric and fully expansion, no accessory muscle used, no operation scar, no spider angiomata, no gynecomastia
    - Palpation: no palpable mass or nodule, no subcutaneous emphysema, no tenderness
    - Ausculation: clear breathing sound
  - Heart:
    - Palpation: no thrill, no heave
    - Percussion: normal heart size
    - Ausculation: regular heart beat, normal S1, S2, no gallop, no murmur
  - Extremities: No deformity, full ranged of motion without limitation, no clubbing finger or nail cyanosis, no pitting edema, no ulcer or pigmentation, no obvious skin lesion, no varicose vein, no bone, joint or muscle tenderness

CRS CONDITION

- Karnofsky performance status: (100) Normal, no complaints, no evidence of disease
- PE of Abdomen:
- __1. Inspection: ok
- __2. Ausculation: ok
- __3. Palapation: ok
- __4. Percussion: ok
- CEA: 3.54 ng/ml
- Imaging Studies:
- Colonoscope report: nil
- Sono , CT of abdomen and chest Films:Finding:

  Sonography evaluation of the abdomen was performed:

  1. Mild fatty liver with a 3.7cm cyst
  2. Right kidney is 10.4cm with a 0.9cm renal stone.
  Left kidney is 10.3cm in size with normal echogenicity.
  No hydronephrosis can be identified.
  3. The gall bladder is normal in size without stone.
  4. The pancreas head and body is normal echogenicity and size. The pancreatic tail is blocked by bowel gas.
  5. The spleen is normal in appearance.

  Impression:
  As above description.
- Figures:
  1. colon & rectum

手術日期及方法

- 手術日期及方法20191018 Low anterior resection with colo-anal anastomosis Ileostomy Laparoscopy

住院治療經過

- After admission, the patient was performed Laparoscopic assisted low anterior resection with end-to-end anastomosis on 10/18. He tolerated well He was discharged on 10/25

合併症

- nil

一般檢查

- 累積報告：

DATE	TIME	ALB	TP	BIL.T	BIL.D	ALKP	AST	ALT	LDH	CHOL	TG	HDL-C	LDL	UA	NH3	AMY
1081018	1940	4.1		0.7	0.2	76	17	14								

DATE	TIME	WBC	RBC	HGB	HCT	MCV	PLT	BLST	NEUT	BAND	SEG	LYM	MON	EOS	BAS
1081018	1941	18400		13.3	39.7		205		83.8			11.0	4.6	0.4	0.2

DATE	TIME	NA	K	CL	CA	BUN	CREAT	Mg	P	CRP	ESR	PCT	LIPASE	LACTATE
1081018	1940	142	4.3	107	8.5	16	1.02							

特殊檢查

病理報告

- pending

其它

出院時情況

- 3.改門診治療

出院指示

藥名	劑量	劑量單位	用法	頻次	數量	數量單位	現況	天數	單位

Ultracet tab	1	TAB	PO	Q6HPRN	20	TAB	TH	5	天
OCAS Harnalidge 0.4 mg	1	TAB	PO	QDAC	11	TAB	TH	11	天
Ulstop tab 20mg	1	TAB	PO	BID	22	TAB	TH	11	天

出院計劃

- 門診日期
  - date on 2019/10/29

主治醫師 2209J 陳志典 / 住院醫師 5657L 林楙時

正式摘要(電子簽章：000000178865398)

W95 / W95

doctorRank:B
